# Fetal derived embryonic-like stem cells improve healing in a large animal flexor tendonitis model

**DOI:** 10.1186/scrt45

**Published:** 2011-01-27

**Authors:** Ashlee E Watts, Amy E Yeager, Oleg V Kopyov, Alan J Nixon

**Affiliations:** 1 Department of Clinical Sciences, Comparative Orthopaedics Laboratory at Cornell University, Ithaca, NY, 14850 USA; 2 Celavet, Inc., Celavie Biosciences, LLC, 2360 Eastman Ave, Suite 101, Oxnard, CA, 93030 USA

## Abstract

**Introduction:**

Tendon injury is a common problem in athletes, with poor tissue regeneration and a high rate of re-injury. Stem cell therapy is an attractive treatment modality as it may induce tissue regeneration rather than tissue repair. Currently, there are no reports on the use of pluripotent cells in a large animal tendon model *in vivo*. We report the use of intra-lesional injection of male, fetal derived embryonic-like stem cells (fdESC) that express Oct-4, Nanog, SSEA4, Tra 1-60, Tra 1-81 and telomerase.

**Methods:**

Tendon injury was induced using a collagenase gel-physical defect model in the mid-metacarpal region of the superficial digital flexor tendon (SDFT) of eight female adult Thoroughbred or Thoroughbred cross horses. Tendon lesions were treated one week later with intra-lesional injection of male derived fdESCs in media or media alone. Therapy was blinded and randomized. Serial ultrasound examinations were performed and final analysis at eight weeks included magnetic resonance imaging (MRI), biochemical assays (total DNA, glycosaminoglycan, collagen), gene expression (*TNC*, *TNMD*, *SCX*, *COL1A1*, *COL3A1*, *COMP*, *DCN*, *MMP1*, *MMP3*, *MMP13, 18S*) and histology. Differences between groups were assessed with Wilcoxon's rank sum test.

**Results:**

Cell survival was demonstrated via the presence of the *SRY *gene in fdESC treated, but not control treated, female SDFT at the end of the trial. There were no differences in tendon matrix specific gene expression or total proteoglycan, collagen or DNA of tendon lesions between groups. Tissue architecture, tendon size, tendon lesion size, and tendon linear fiber pattern were significantly improved on histologic sections and ultrasound in the fdESC treated tendons.

**Conclusions:**

Such profound structural effects lend further support to the notion that pluripotent stem cells can effect musculoskeletal regeneration, rather than repair, even without *in vitro *lineage specific differentiation. Further investigation into the safety of pluripotent cellular therapy as well as the mechanisms by which repair was improved seem warranted.

## Introduction

Overstrain injuries to weight bearing tendons are common in human [1,2] and equine [3,4] athletes with many similarities between the two [5,6]. Commonly injured tendons include the Achilles tendon in humans and the superficial digital flexor tendon (SDFT) in the horse. These injuries are predominantly degenerative in nature, slow to heal, and rarely regain their original strength and elasticity [5,7]. This inferior healing leads to prolonged rehabilitation times and a high re-injury rate [1,7]. Despite improvements in early detection, advances in rehabilitation techniques, and numerous new biologic and cellular therapies, a consistently successful treatment regimen has yet to be developed [5,7-9].

Due to the low cellularlity and low mitotic activity of tendons, intrinsic tendon repair is largely performed by cells of the endotenon and epitenon with some proliferation of tenocytes at the perimeter of the lesion [10]. Extrinsic repair may be influenced by microvascular pericytes and endothelial cells associated with blood vessels [11]. The paucity of an appropriate cell for tendon regeneration may explain the prolonged healing times, disorganized scar tissue formation, and inferior mechanical properties of healed tendons [12]. This fact has led to an interest in cellular therapies for tendon injury that may recapitulate tendon development, resulting in tendon regeneration [13]. Adult derived mesenchymal stromal (stem) cells (MSCs), the multipotent precursor cells of connective tissues, have been used toward this goal experimentally in rats [14,15], rabbits [16], horses [17-19] and sheep [20], and empirically for clinical tendon injury in horses [21,22] for the past several years. Despite significant improvements to re-injury rates [21], and minor improvements to histologic architecture [17,18], MSCs have not induced the degree of tendon regeneration that is seen in injured fetal tendon [23]. Utilizing a cell line with greater plasticity and proliferative capacity than adult multipotent MSCs, may better contribute to tendon regeneration [24]. To date, there have been no studies exploring the use of pluripotent cells in the treatment of tendon injury in a large animal model.

Currently, there is no successful method for isolation of equine ESCs [25]. In order to avoid necessary genetic manipulations of induced pluripotent stem (iPS) cells [26-29], an allogenic cell line (OK-100™; Celavet, Inc., Oxnard, CA, USA) derived from equine fetal tissue and induced to express markers of pluripotency through culture conditions was utilized. The objective of this study was to examine the effect of a pluripotent cell versus placebo control on tendon healing in a large animal model of experimental tendon injury.

## Materials and methods

### Animals

Eight adult female Thoroughbred (*n *= 7) or Thoroughbred cross (*n *= 1) horses, ranging in age from three to seven years, without clinical or ultrasonographic evidence of tendon injury were used. All horses had undergone rigorous athletic training prior to inclusion in the study. Horses were housed separately, in box stalls, and allowed to acclimate to the environment for ≥2 weeks prior to study initiation. All invasive procedures were performed by experienced board certified veterinary surgeons. This study was approved by and performed according to guidelines of the university's Institutional Animal Care and Use Committee.

### Cell isolation, culture

To allow for testing of a pluripotent cell, a commercially available cell line (OK-100™) was used. Briefly, the cell line was prepared from an equine fetus obtained early in gestation by uterine flushing. Fetal tissue, specifically brain, spinal cord, liver and heart, was dissected and each organ was separately minced with microscissors and then triturated with Pasteur pipettes until a single cell suspension was obtained. Cells were cultured in non-adherent culture flasks in serum free culture medium of Eagle's essential medium (Lonza RR116254, Walkersville, MD, USA) supplemented with B27 (Invitrogen 17504, Carlsbad, CA, USA), calcium chloride (Fisher Scientific, Pittsburgh, PA, USA), Epidermal Growth Factor (Peprotech 100-15, Rocky Hill, NJ, USA), Basic Fibroblast Growth Factor (Peprotech 100-18B, Rocky Hill, NJ, USA), Transforming Growth Factor Alpha (Peprotech 100-16A, Rocky Hill, NJ, USA), Leukemia Inhibitory Factor (Millipore LIF1010, Temecula, CA, USA), L-Glutamine (Invitrogen 25030, Carlsbad, CA, USA), and a nitrogen supplement (Invitrogen 17502, Carlsbad, CA, USA) all added at proprietary concentrations (patent 7632681Celavie Biosciences, LLC, Reading, PA, USA). Cells were passaged approximately weekly by centrifugation for five to six months. Four days after each passage, 4 mL of fresh culture medium was added to culture flasks. Beginning at three months, an aliquot of cells was tested for markers of pluripotency and this was repeated monthly until cells were >70% positive for Oct-4, nanog, telomerase, SSEA4, Tra 1-60 and Tra 1-81 and 100% negative for major histocompatibility complex proteins I and II and p53 (data not shown). Once this was confirmed, chromosomal microarray was used to confirm that genomic deletions or duplications had not occurred during the culture period (data not shown).

### Study design

**Figure 1:**
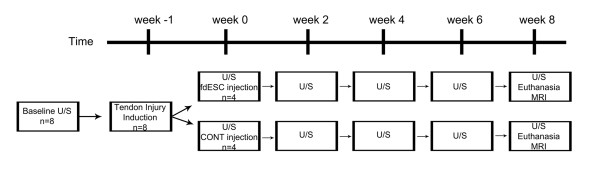
**Study timeline**. CONT, placebo control; fdESC, fetal derived embryonic-like stem cells; MRI, magnetic resonance imaging; U/S, ultrasound.

The study consisted of two randomly assigned groups: group A (stem cell treated tendons; *n *= 4; fdESC) and group B (placebo treated tendons; *n *= 4; CONT). One week after tendon injury, treatment injections were performed. Ultrasound examinations were performed every two weeks, thereafter. Eight weeks after treatment injection, animals were euthanized, magnetic resonance imaging was performed and tissues were collected (Figure 1). Other than an off-site control officer, all investigators were blinded to treatment group identification until the study was completed and all assays were performed. Treatment group (A or B) was revealed for statistical analysis. Once all analyses were completed, treatment group identification (fdESC or CONT) was disclosed.

### Tendon injury induction

Collagenase-induced lesions were created in the tensile region of the superficial digital flexor tendon (SDFT) of one randomly selected forelimb using filter sterilized bacterial collagenase type I (Sigma, St. Louis, MO, USA). Forelimb selection (left or right) was made by a coin toss for the first horse and then alternated for each subsequent horse. Thirteen hundred units of collagenase was delivered as a gel to a columnar physical defect centered within the tensile region of the SDFT tendon (16 to 18 cm distal to the accessory carpal bone; DACB) using a 16 gauge 8.89 cm Weiss Epidural needle with a Tuohy tip (BD, Franklin Lakes, NJ, USA) inserted under ultrasonographic guidance, as modified (Watts AE, Yeager AE and Nixon AJ, Submitted) from previous descriptions [17,30,31]. The study forelimb was bandaged.

### Treatment injections for cell transplant

One week post collagenase tendon injury induction (t = 0 weeks), tendon lesions were treated with two ultrasonographically guided intra-lesional treatment injections. The day before treatment injection, three million fdESCs, resuspended in 1.5 mL culture media, or 1.5 ml culture media alone for placebo injection, were packaged in 2 mL coded cryovials and transported overnight to the animal facility. For injection, local anesthesia at the sites of needle insertion was achieved with 1 to 3 ml lidocaine (20 mg/ml) in the subcutaneous tissue and standing sedation (xylazine 0.5 mg/kg IV and butorphanol 0.01 mg/kg IV). Treatment injection to the lesion was performed with 25 gauge needle entry at 16 and 18 cm DACB, directed from palmarolateral to dorsomedial. At the time of treatment injection, horses were given anti-inflammatory medications (phenylbutazone 4.4 mg/kg bwt IV and dexamethasone 0.04 mg/kg bwt IV). Non-steroidal anti-inflammatory medication was continued for two days (phenylbutazone 2.2 mg/kg PO q24 h). Horses were confined individually to box stalls for the duration of the study and their treated forelimb was bandaged for the first five weeks after lesion induction.

### Lameness/reaction data

Physical examination was performed and vital parameters were recorded every 12 hours, and bandage changes (up to five weeks after lesion induction) and limb examinations were performed daily throughout the study. Lameness at a walk was assessed every six hours for three days following lesion induction (t = -1 weeks) and treatment injection (t = 0 weeks) and every 12 hours throughout the remainder of the study.

### Ultrasound

Ultrasound examinations were performed prior to admission to the study (baseline) and at t = 0, 2, 4, 6, and 8 weeks after treatment injection. Ultrasound imaging was performed by a board-certified veterinary radiologist (AEY) using a real-time ultrasound machine (iU22, Philips Healthcare, Amsterdam, The Netherlands) equipped with broad-band technology and linear probes of high frequency (5 to 12 MHz). A template was used to ensure accurate repetition of tissue gain settings, focus, and depth of tissue penetration. Longitudinal and transverse ultrasound images were acquired and tendon cross-sectional area (TCSA), lesion cross-sectional area (LCSA), and a longitudinal linear fiber pattern score were measured by the same ultrasonographer at 16 cm DACB. The LCSA as a percentage of TCSA was calculated for relative lesion cross-sectional area (RLCSA).

### Tissue harvest and magnetic resonance imaging

Horses were euthanized by pentobarbital overdose at eight weeks post treatment injection and their treated forelimb was collected for immediate magnetic resonance imaging (MRI) with a 0.3 Tesla magnet (Vet MR, Esaote, Genova, Italy). Limbs were positioned in extension for T1 and T2 image acquisition in the sagittal and transverse planes. Measurements of TCSA and LCSA based upon the area of hyperintense signal were made at 16 cm DACB on T1 images. The lesion was also graded for the intensity of increased MR signal on T1 images (0 = normal; 1 = mild increase; 2 = moderate increase; 3 = marked increase; 4 = intense increase, equal to bone marrow signal).

Following MRI, limbs were dissected under RNase free conditions and samples were collected from the center of the tendon lesion at 16 cm DACB extending into the surrounding normal tendon. Samples were snap-frozen in liquid nitrogen, pulverized in a freezer-mill and stored at -80°C until use, or fixed in 4% paraformaldehyde at 4°C for 72 hours.

### RNA and DNA isolation and qPCR

Total cellular RNA was isolated from pulverized tissue using a commercially available RNA extraction kit (PerfectPure RNA Fibrous Tissue Kit, 5 Prime, Gaithersburg, MD, USA). Genomic DNA was isolated from pulverized tissue using a commercially available genomic DNA extraction kit (PureLink Genomic DNA kit, Invitrogen, Carlsbad, CA, USA). All qPCR probes and primers were designed using equine specific sequences published in Genbank (Additional File 1 Table S1). Genomic DNA was removed from RNA samples prior to PCR, by DNase I digestion. RNA and genomic DNA quality was assessed by spectrophotometry at 260:280 nm and by 1% agarose gel electrophoresis (data not shown). Total RNA was reverse transcribed and amplified using the One-Step RTPCR technique and the ABI PRISM 7900HT Sequence Detection System (Applied Biosystems, Life Technologies, Carlsbad, CA, USA). All samples for each molecule were assessed at the same time on the same qPCR plate to minimize variation. The qPCR program included reverse transcription at 48°C for 30 minutes and denaturing at 95°C for 10 minutes, followed by 40 cycles of 90°C for 15 seconds and 60°C for 1 minute. For gene expression, each well of the qPCR plate was loaded with 10 ng of RNA in 20 μl. For DNA, several different loading concentrations were utilized, including 10, 25, 50, 100 and 200 ng of DNA per well and the number of melting and annealing cycles was increased from 40 to 55. Other than *18S*, a standard curve was generated from equine specific plasmid DNA for each gene at known concentrations to allow copy number estimation. The primers and dual-labeled fluorescent probe (6-FAM as the 5' label (reporter dye) and TAMRA as the 3' label (quenching dye)) were designed using Primer Express Software version 2.0b8a (Applied Biosystems) using equine specific sequences published in Genbank. All samples were run in triplicate on the qPCR plate and total copy number per ng of RNA of each gene was obtained from a standard curve and normalized to *18 S *gene expression for collagen types I and III (*COL1A1*, *COL3A1*), decorin (*DCN*), cartilage oligomeric matrix protein (*COMP*), tenascin-C (*TNC*), tenomodulin (*TNMD*), scleraxis (*SCX*) and matrix metalloproteinases-1, 3 and 13 (*MMP1*, *MMP3*, *MMP13*).

### Biochemical analysis

Pulverized tendon samples were lyophilized for biochemical assays. For total glycosaminoglycan and total DNA assay, samples were digested in papain (1 mL papain (0.5 mg/ml)/10 mg lyophilized tendon) at 65°C for 4 and 24 hours, respectively. The samples were mixed with dimethylmethylene blue dye for glycosaminoglycan quantification by colorimetric assay [32] and bisbenzimide compound for DNA quantification by fluorometric assay [33] in triplicate aliquots. Total soluble collagen content was determined in triplicate aliquots using the Sircol Assay (Biocolor LTD., Carrickfergus, Northern Ireland, UK) according to the manufacturer's directions for pepsin soluble collagens with modifications as previously described [34].

### Histology

Fixed longitudinal tissue sections were softened in 4% phenol in 70% alcohol for five days [31,35] embedded in paraffin, sectioned and stained with hematoxylin and eosin (H&E) or Picrosirius Red and examined under white light and polarized light microscopy. Sections were also prepared for fluorescent *in situ *hybridization with probes produced using nick translation against genomic SRY, [GenBank: EU599187.1] [36]. All slides were examined by two blinded investigators (AJN and AEW), using a calibrated reticule to sequentially examine across and down the entire tendon section, under low power and high power where appropriate for cell detail, to derive a complete histologic impression. For fluorescent *in situ *hybridization, slides were characterized as being positive or negative for probe hybridization. For routine histology, scores were assigned for two sections from each tendon (proximal and distal within the lesion, centered at 16 DACB). All tendon parameters were scored from l (normal) to 4 (severe changes) for: tenocyte shape, tenocyte density, free hemorrhage, neovascularization, perivascular cuffing, collagen fiber linearity, collagen fiber uniformity and polarized light crimping. Scores from both segments (proximal and distal) and both observers were averaged. This grading scheme expands on previously described systems which utilize an eight-parameter, four-point score [37-39].

### Statistical analyses

Numerical data were tested for normality. Once a non-normal distribution was confirmed, non-parametric statistics were utilized. Differences between treatment groups were tested using Wilcoxon's rank sum analysis. For ultrasound and MRI data where we expected fdESC treated tendons to be smaller, with higher fiber pattern scores and less tissue signal, a one-sided test was utilized. For all other data, a two-sided test was utilized. Repeated measures analysis was performed within each group on ultrasound data at differing time points using Wilcoxon's signed rank tests. Except for repeated measures analysis, all ultrasound data were normalized as a percent of the baseline measurement prior to lesion induction (baseline) or the score of the lesion on the first day of treatment at t = 0 weeks. Gene expression, histologic scores, MRI measurements, and biochemical data were reported as a median and 95% confidence interval. Ultrasound data from all time points were reported with box plots, as a median and quartiles. To test for differences in post-treatment lameness (yes/no), a Fisher's exact test was used. For all tests, Statistix 9 software (Analytical Software, Tallahassee, FL, USA) was used and significance was set at *P *< 0.05.

## Results

### Lameness/reaction data

The day of treatment injection, two fdESC treated horses had mild lameness at a walk prior to treatment injection and no lameness was appreciable in any of the CONT treated horses. This proportion was not different (one-tailed *P *= 0.21). In the days following treatment injection, the same two fdESC treated horses that were lame prior to injection, developed increased lameness and required extension of analgesic/anti-inflammatory medication (phenylbutazone) duration by six and seven days beyond routine therapy and two CONT treated horses developed increased lameness, only one of which required extension of analgesic/anti-inflammatory medication (phenylbutazone) duration by five days. No lameness was noted at a walk in either group throughout the remainder of the study (t = 1 to 8 weeks).

### Ultrasound data

**Figure 2:**
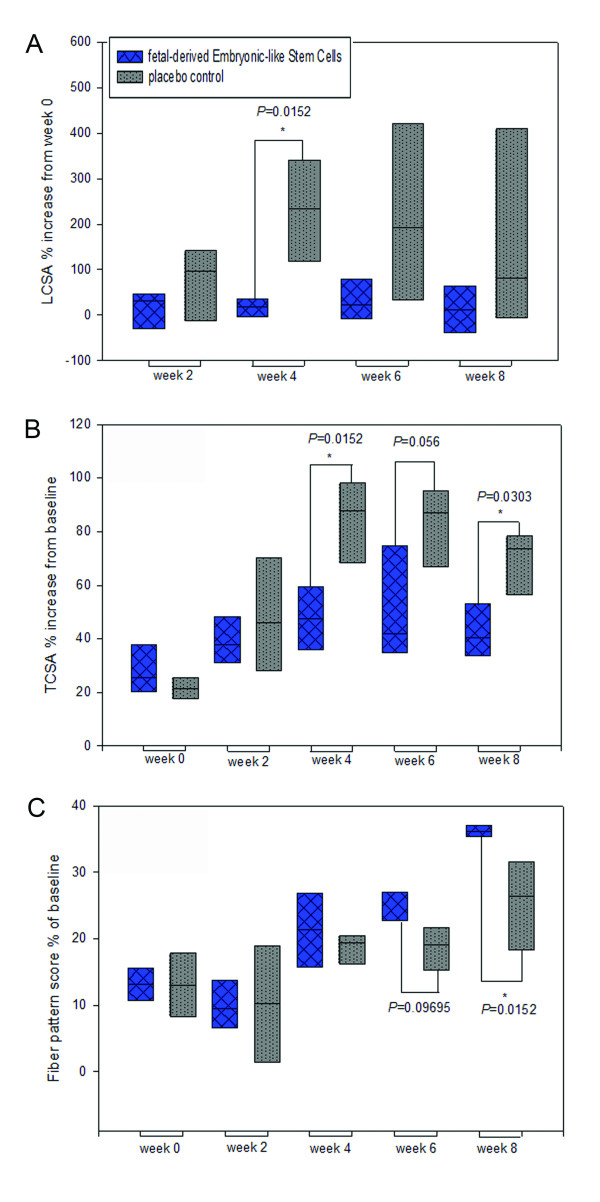
**Ultrasound measurements**. Normalized **(A) **lesion and **(B) **tendon cross-sectional area (CSA) and **(C) **linear fiber pattern score for fetal-derived Embryonic-like Stem Cells (fdESC) and placebo control (CONT) treated tendons at time points post treatment injection in weeks. Asterisks mark significantly lower (A, B) or significantly higher (C) values for fdESC than CONT treated tendons (one-tailed *P *< 0.05).

Normalized lesion CSA was significantly lower in fdESC treated tendons compared to CONT tendons at t = 4 weeks (one-tailed *P *= 0.02) and showed a trend for smaller CSA at t = 8 weeks (one-tailed *P *= 0.1; Figure 2A). Normalized tendon CSA was significantly lower in fdESC treated tendons compared to CONT tendons at t = 4 (one-tailed *P *= 0.02) and eight weeks (one-tailed *P *= 0.03) and showed a trend for smaller CSA at t = 6 weeks (one-tailed *P *= 0.06; Figure 2B). Normalized relative lesion CSA was significantly lower in fdESC tendons compared to CONT tendons at t = 4 weeks (one-tailed *P *= 0.02). Normalized fiber pattern score, was significantly higher (better) for fdESC treated tendons at t = 8 weeks (one-tailed *P *= 0.02; Figure 2C).

On paired analysis, within the fdESC treated tendons (one-tailed *P *= 0.05) but not the CONT treated tendons (one-tailed *P *= 0.1), there was a trend for higher (better) fiber pattern score at t = 8 weeks versus t = 0 weeks.

**Figure 3:**
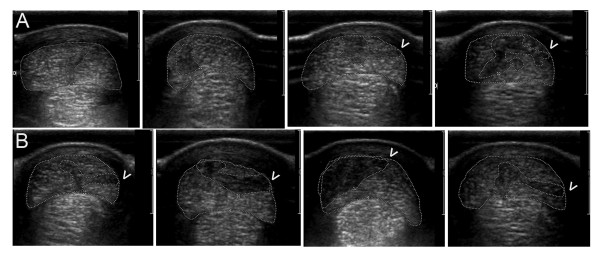
**Transverse ultrasound images**. Images were made 16 cm distal to the accessory carpal bone, eight weeks post treatment with **A) **fetal-derived Embryonic-like Stem Cells or **B) **placebo control injections. Lateral is to the right. Dotted lines outline the superficial digital flexor tendon and lesion. Arrowheads identify remaining treatment injection needle tracts.

From two weeks after treatment injections (t = 2 weeks) needle tracts were visible ultrasonographically at 16 and 18 cm distal to the accessory carpal bone in all CONT treated tendons at all time-points. During the same period (t = 2 to 8 weeks), needle tracts were visible, but difficult to discern in two fdESC treated tendons. At the completion of the study, needle tracts remained visible in all CONT tendons and only two fdESC tendons (Figure 3). This proportion was not statistically different (one-tailed *P *= 0.2).

### Magnetic resonance imaging

**Figure 4:**
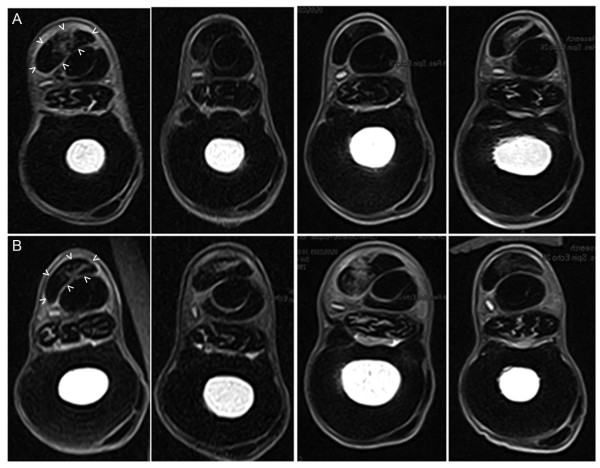
**Transverse T1 MR images**. Images were made at 16 cm distal to the accessory carpal bone, post mortem, eight weeks after treatment injection with **A) **fetal derived Embryonic-like Stem Cells and **B) **placebo control. Lateral is to the right. Arrow-heads outline the treated tendon in the first image of each group.

Tendon lesions were identified by increased signal intensity in all tendons (Figure 4). Tendon CSA made on MR images were not different between groups (Table 1). There was a strong trend of reduced relative CSA and reduced lesion CSA of fdESC treated tendons compared to that of CONT tendons (one-tailed *P *= 0.05 and *P *= 0.06, respectively; Table 1). Lesion signal intensity scores for fdESC tendons were lower (more normal) but this was not statistically significant (Table 1).

**Table 1:** Tendon and lesion measurements based on transverse T1-weighted MRI at eight weeks

	Fetal-derived Embryonic-like Stem Cell treated tendon		Placebo control treated tendon		
			
	Median	95% Confidence Interval	Median	95% Confidence Interval	One-tailed *P*
Relative CSA	0.25	0.1566 to 0.3573	0.4	0.1654 to 0.7040	0.06
Lesion CSA	0.34	0.1463 to 0.5407	0.53	0.1069 to 1.1942	0.06
Tendon CSA	1.28	0.9489 to 1.6776	2.0	1.0088 to 1.9261	0.3
Signal Intensity	0.5	-0.7 to 2.3	2.0	0.2 to 4.3	0.07

Measurements of the superficial digital flexor tendon and lesion were made 16 cm distal to the accessory carpal bone. Tendon, lesion and relative lesion cross-sectional area (CSA) and signal intensity were assessed.

### Gross dissection

No peritendinous adhesions were noted during dissection in either group. Once dissected free, tendons from both groups were visibly enlarged, centered at 16 cm DACB, and had minimal peri-tendinous reaction (Additional File 2, Figure S1). Focal pink discoloration was present superficially in all tendons proximally, at the site of needle insertion for tendon injury induction and distolaterally, at the sites for treatment injection (Additional File 2, Figure S1). Although no scores were assigned, fdESC treated tendons appeared smaller at 16 cm DACB, had less peri-tendinous reaction and treatment injection sites were less obvious. On cut section, lesions were hemorrhagic, glistened and bulged from the cut surface in all tendons (Additional File 2, Figure S1).

### Quantitative PCR

Good quality RNA and DNA was obtained from all samples (data not shown). RNA concentrations from 80 mg of tissue (wet weight) was not different between groups (2-tailed *P-*value = 0.2; fdESC median 517 ng/μl, range 318 to 670 ng/μl; CONT median 370 ng/μl, range 293 to 513 ng/μl). DNA concentration from 25 mg of tissue (wet weight) was significantly lower in fdESC versus CONT samples (2-tailed *P *= 0.04; fdESC median 31 ng/μl; range 27 to 35 ng/μl; CONT median 41 ng/μl; range 34 to 49 ng/μl). There were no significant differences in anabolic (*COL1A1*, *COL3A1*, *DCN*, *TNC *or *COMP*), catabolic (*MMP1*, *MMP3 *or *MMP13*) or phenotypic (*SCX*, *TNMD*) gene expression between groups (Additional File 3, Table S2). There was no amplification of SRY above the level of no template controls, in either group, at any of the tested loading concentrations.

### Biochemical analyses

There were no significant differences in DNA (two-tailed *P-*value = 0.09), glycosaminoglycan, or total collagen content between fdESC tendons and CONT tendons (Additional File 3, Table S2).

### Histology

**Figure 5:**
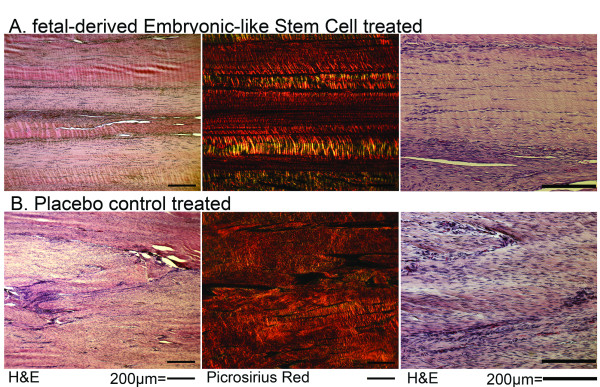
**Longitudinal histology**. This figure shows 50× and 200× magnification of longitudinal sections of superficial digital flexor tendon stained with H&E and Picrosirius Red for **A) **fetal-derived Embryonic-like stem cell treated tendon and **B) **placebo control treated tendon. Picrosirius images shown under polarized light. Bars = 200 μm.

**Figure 6:**
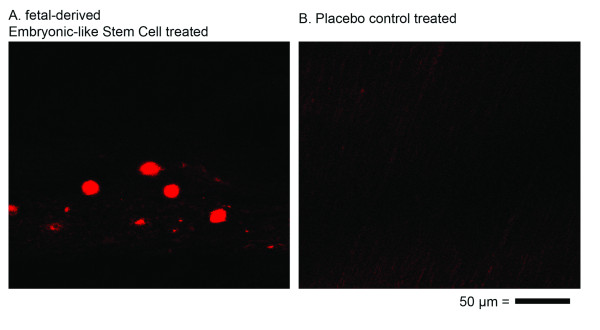
***In situ *hybridization longitudinal histology**. 400× magnification of *in-situ *hybridization against genomic SRY in **A) **fetal-derived Embryonic-like stem cell treated tendon and **B) **placebo control treated tendon. Bar = 50 μm.

Cumulative histology scores were significantly different (more normal) for fdESC treated tendons compared to CONT tendons (Figure 5; Table 2). Several individual parameters were significantly different (more normal) in fdESC treated tendons compared to controls (Table 2). No individual parameters were higher (less normal) in fdESC treated tendons compared to CONT. *In situ *hybridization with probes against equine SRY demonstrated the occasional persistence of injected fdESC cells in all fdESC treated tendons but not in the CONT tendons (Figure 6).

**Table 2:** Histologic scoring

Tendon parameter	Fetal-derived embryonic-like stem cell treated tendon	Placebo control treated tendon	Two-tailed *P*
Cell shape	1.0 (0.9 to 1.3)	2.0 (1.8 to 2.3)*	<0.0001
Cell density	1.0 (2.0 to 2.0)	2.75 (2.3 to 3.0)*	0.0002
Free hemorrhage	1.0 (0.9 to 1.7)	1.75 (1.1 to 2.4)	0.1
Neo-vascularization	2.0 (1.4 to 2.2)	2.0 (1.2 to 2.3)	0.7
Perivascular cuffing	1.5 (1.1 to 2.0)	1.0 (0.83 to 1.4)	0.1
Collagen linearity	2.0 (1.3 to 2.1)	2.75 (2.4 to 3.0)*	<0.0001
Collagen uniformity	2.0 (1.4 to 2.1)	3.0 (2.8 to 3.1)*	<0.0001
Polarized crimping	1.5 (1.2 to 2.1)	3.0 (2.7 to 3.3)*	<0.0001
Epitenon thickening	2.3 (2.0 to 2.6)	3.0 (2.6 to 3.5)*	0.0009

**Cumulative Score**	15.4 (12.6 to 17.8)	20.8 (19.0 to 23.0)*	<0.0001

Histologic scores for fetal-derived embryonic-like stem cell treated tendon and placebo control treated tendon reported as median (95% confidence interval). Significant differences between treatment groups are marked by an asterisk (two-tailed *P *< 0.05) and the two-tailed *P-*values are listed.

## Discussion

This blinded, placebo-controlled, large animal, short-term (eight-week) experiment revealed substantial and clinically relevant improvement in the healing of tendon injury after intra-lesional injection of pluripotent stem cells. Such dramatic architectural improvements have not been shown previously with any treatment modality, including the multipotent, autogenous MSC or ADSC [17,19].

Despite widespread use of the collagenase enzymatic degradation model of tendon injury to test various therapeutics, including fat derived and bone marrow derived autogenous MSCs, no large animal study to date has demonstrated measureable differences in any parameter, other than small improvements in histologic grading [17,19]. In the study reported here, fdESC treated tendons had significant structural improvement on MRI and ultrasound, compared to CONT treated tendons; fdESC treated tendons were smaller and had smaller lesions with better lesion fill and greater return to more normal linear fiber pattern. In clinical equine tendon injury, other than severity of the initial lesion, the development of normal fiber pattern is the single most predictive measure of successful long term outcome [40]. Therefore, the improved linear fiber pattern scores in fdESC treated tendons demonstrate significant and clinically relevant superior healing in the fdESC group, and suggest at least faster injury resolution, if not an improvement in long term outcome. Although there is little available data on the MRI appearance of healing tendons, it is known experimentally that reduced lesion signal intensity is correlated with tendon mechanical recovery [41] and reduction in pain [42]. Therefore, the trend toward reduced MR signal intensity (one-tailed *P *= 0.07) and trend for reduced lesion and relative CSA on MRI (one-tailed *P *= 0.06) in fdESC treated tendons corroborates better tendon injury resolution.

In a clinical report of the use of MSCs for flexor tendon injury in horses, lesions resolved following treatment; however, needle tracts from treatment injections remained visible on all follow-up ultrasound examinations [21]. Therefore, the inability to find needle tracts in two fdESC treated tendons and difficulty discerning needle tracts in the other two fdESC treated tendons, although not statistically significant, is remarkable, and may represent a major change in the lesion environment, occurring as soon as two weeks after the treatment date. Additionally, during gross examination at eight weeks, needle insertion sites for the treatment injection were grossly less obvious in fdESC treated tendons (Additional File 2, Figure S1).

Despite its many similarities, the collagenase model of tendon injury does not totally mimic the insidious and degenerate etiopathogenesis of many forms of naturally occurring flexor tendon injury in man. However, the clinical relevance of this model to the final acute disruption after months or years of chronic tendon injury, is supported by the evaluation of gross, biochemical and histopathological changes, clinical signs, mechanical characteristics, and MRI and ultrasonographic findings following the induction of injury [43,44]. Additionally, the collagenase gel model allows the generation of a homogenous tendon lesion in a controlled group of animals and, therefore, improved ability to detect differences between treated and control arms of the study. The equine mid-metacarpal SDFT is a large, weight bearing tendon that is easily accessible, is not confined to a synovial sheath, and in the equine athlete is commonly affected by naturally occurring over-stretch tendon injury compounded on previous microfiber disruption, similar to tendinopathies of the human Achilles tendon [5,6]. Another major benefit of this model in testing cellular therapies for tissue regeneration is the creation of a confined lesion, surrounded by normal tendon, which is a common feature of Achilles tendon (human) and SDFT (equine) injury. This allows the direct and focal application of cellular therapies to a closed environment, where cellular differentiation can occur through naturally occurring biochemical cues, biomechanical forces, growth factors, and adjacent cell signaling. This is in direct contrast to rodent and small animal models where acute surgical transection of flexor tendons is utilized and tendons are of insufficient size to allow confined and directed focal therapy [45]. However, a limitation of this model is the inability to test therapies in a large number of animals, resulting in a study that may be underpowered. This is due to the significant cost of housing, buying and caring for these animals and the strong emotional and ethical considerations in their use and sacrifice [46].

An additional limitation to this study was the short term end-point which was selected to assess the acute effects of the cells on tendon cell population, organization, and behavior. Analysis at eight weeks was selected as it was the earliest time-point that structurally organized tissues, and, therefore, potential differences, were expected to be detectable. Despite the small group sizes (*n *= 4), several parameters were significantly different between groups and a few parameters were different as early as four weeks after treatment injection, providing strong evidence for improved healing due to fdESC therapy. However, the lack of significant differences in parameters such as total DNA and gene expression should be viewed with caution, as a higher powered study may have better identified differences if they existed. A final limitation of this model was the inability to determine the mechanism by which fdESC injection improved healing, that is, trophic factors, cell replacement or other mechanisms.

Both collagen type I and collagen type III are upregulated in tendons following injury, with increased gene expression (*COL1A1 *and *COL3A1*) and protein content (type III) [47]. Despite its upregulation in healing tendon, collagen type III content remains low compared to collagen type I [48] and its exact role in tendon healing is largely unknown. Certainly lower collagen type I to collagen type III ratios indicate scar tissue repair rather than tendon regeneration; however, collagen ratios generated during tendon tissue regeneration rather than repair are not strictly known. In this study, there were no differences in total collagen content and ratios of *COL1A1:COL3A1 *gene expression between fdESC and CONT treated tendons, which may reflect the importance of tissue organization during healing rather than tendon tissue constituents. The improved collagen fiber diameter and alignment seen histologically, as well as improved linear fiber pattern seen ultrasonographically, and reduced MR signal intensity, suggest that although total collagen content is not different, there are improved collagen characteristics in the fdESC treated tendons.

While compositional parameters such as gene expression and proteoglycan and collagen content accurately reflect constituents of the healing tendon, they do little to measure the organization of the tendon. Structural assessment data indicated fdESC treated tendons had better histologic scores and improved MRI and ultrasound measurements and scores, compared to CONT treated tendons. These findings, and the lack of significant differences in biochemical parameters (DNA, glycosaminoglycan, and total collagen content) and gene expression data between fdESC and CONT, suggest that the predominant effect of fdESCs on tendon healing is through tendon structural organization rather than cell numbers or anabolic gene expression. Alternatively, localized changes, especially in gene expression, could have been missed due to total homogenization of the tissue samples, leading to incorporation of enough surrounding normal tendon to mask any significant differences between treatment groups [49]. However, this seems unlikely given the size of equine tendons, allowing careful collection of lesion tissue and immediately adjacent tendon tissue. Additionally, tendon healing is a slow process, normally taking up to 18 months to occur. Therefore, this study is likely to have fallen short of the ultimate result, and it is possible that gene expression differences would be apparent in longer term trials.

Although there was no statistically significant difference in total DNA content determined by fluorometric assay, there was a trend toward lower total DNA content in fdESC treated tendons (two-sided *P *= 0.09). This could be interpreted as the failure of injected fdESC cells to persist within the lesion. Alternatively, we suggest that injected fdESC cells induced tendon regeneration leading to fewer cells and the accumulation of more normal, less cellular, tendon matrix, with fewer but more functional tenocytes. Significantly reduced concentrations of DNA isolated during genomic DNA preparations from fdESC tendons compared to CONT tendons, and a trend toward reduced total DNA content during quantitative fluorometric assay, corroborate the histologic findings indicating fdESC tendons were less cellular.

Although MSCs modulate immune function [50], no such effects have been reported for pluripotent stem cells, and the risk of using allogenic pluripotent stem cells is poorly defined. In this study, no adverse effects due to the use of allogenic cells were expected because the cell line did not express major histocompatibility proteins, and none were noted. The reduced cell density, improved cell shape, lack of inflammatory infiltrate or change in vascularity on histologic sections, minimal peri-tendinous reaction grossly, lack of differences in post treatment injection lameness, and reduced MR signal suggest that there was minimal reaction to allogenic fdESCs, despite lack of immunosuppressant therapy. Additionally, the risk for teratoma formation in immune competent animals with transplant of a pluripotent cell line is unknown. A teratoma assay was not performed for the cell line OK100™. However, teratoma formation was not seen in other fetal-derived cell lines used for human neurodegenerative disease trials [51]. Additionally, the possibility of teratoma formation seems unlikely given that the cells are not true embryonic stem cells as they are negative for alkaline phosphatase staining of colonies and are derived from fetal tissue, rather than embryos (data not shown; Celavet, Inc.). Although there was no evidence of teratoma formation, it is important to note that transplant cell numbers were relatively low and this was a short term experiment using a small number of animals. Given the small number of horses used in this study, safety should be confirmed in a larger number of animals, longer term.

The use of male derived fdESCs in female recipient horses was utilized to identify cell transplantation persistence without genetic or cellular modification. Cellular persistence was documented with *in situ *hybridization on histologic sections, although it was an unusual event, and was not corroborated by PCR amplification of the SRY gene. Low cell survival may be due to the immunogenicity of male cells in female animals with chronic rejection occurring secondary to antibody responses to Y-chromosome encoded minor histocompatibility antigens preventing long-term engraftment [52,53]. It is possible that injecting genetically or membrane dye labeled same-sex cells would better gauge and allow for cell survival in follow-up studies [54]. Finally, this study does not define whether the fdESCs had an effect through exogenous cell replacement, local cytokine modulation, immune modulation, or the stimulation of trophic factor synthesis. Certainly, the rarity of long term cell survival would suggest that it is less likely exogenous cell replacement, and may be one, or all of the latter factors.

## Conclusions

In conclusion, these findings support the efficacy of pluripotent stem cells for the treatment of tendon injury. Despite low long term survival of injected cells, intralesional injection with fdESCs resulted in significantly better ultrasonographic measurements and scores, significantly better histological scores and a strong trend for improved MRI parameters. Such profound structural improvements to healing tendon in this short term large animal study lend further support to the notion that pluripotent stem cells can effect musculoskeletal regeneration, rather than repair, even without *in vitro *lineage specific differentiation. Further investigation into the safety of pluripotent cellular therapy as it relates to karyotypic stability, maintained cellular localization and avoidance of uncontrolled differentiation, as well as the mechanisms by which repair was improved, need to be determined.

## Abbreviations

ADSC: adipose derived stem cell; *COL1A1*: collagen type I; *COL3A1*: collagen type III; *COMP*: cartilage oligomeric matrix protein; CONT: control; DCN: decorin; DACB: distal to the accessory carpal bone; fdESC: fetal derived embryonic-like stem cells; iPS: induced pluripotent stem; LCSA: lesion cross-sectional area; *MMP1*: matrix metalloproteinases-1; *MMP3*: matrix metalloproteinase-3; *MMP13*: matrix metalloproteinase-13; MRI: magnetic resonance imaging; MSCs: mesenchymal stromal (stem) cells; RLCSA: relative lesion cross-sectional area; *SCX*: scleraxis; SDFT: superficial digital flexor tendon; TCSA: tendon cross-sectional area; *TNC*: tenascin-C; *TNMD*: tenomodulin; *SCX*: scleraxis.

## Competing interests

OVK is a full time employee of Celavie Biosciences, LLC, the company that provided the cell line (OK100™), and a patent holder on the cell line OK-100, studied in this report. OVK also holds stock in the company Celavie Biosciences. LLC. AJN and Cornell University was the recipient of a grant from Celavet, Inc. to fund this study. All other authors declare that they have no competing interests.

## Authors' contributions

AEW, OVK and AJN designed the study. OVK carried out all the *in vitro *cell preparation for OK100™. AEW, AEY and AJN carried out all *in vivo *work and imaging (ultrasound, MRI). AEW carried out all the post mortem tissue collections and laboratory assays. AEW and AJN carried out all histologic assessments. AEW and AJN were responsible for writing the manuscript and all authors approved the manuscript.

## Authors' information

AEW is a large-animal surgeon completing a PhD in AJN's lab at Cornell University. The focus of the PhD is on stem cells as they relate to musculoskeletal repair.

AEY is a board certified veterinary radiologist with specific expertise in ultrasonography at Cornell University and has extensive experience evaluating tendon injury, both experimental and clinical.

OVK is an MD, PhD and is employed by Celavie Biosciences, LLC, as its chief science officer and is interested in fetal-derived stem cells and their ability to influence neurodegenerative and musculoskeletal disorders. OVK has extensive experience in the use of fetal-derived stem cells (similar cells to those described in this manuscript) for the treatment of Parkinson's and Huntington's disease, both experimentally and in clinical patients.

AJN is a professor and clinician scientist at Cornell University, and director of a lab interested in stem cells and musculoskeletal injury. AJN has extensive experience with modeling equine tendon injury and in local delivery of cell-based therapies for musculoskeletal injury, both tendon and cartilage.

## Supplementary Material

Additional File 1 **rt-PCR primer and probe sequences**. Sequences (5' to 3') for forward and reverse primers and probes used in quantitative PCR. Sequences were selected from equine specific sequences published in GenBank. Click here for file

Additional File 2 **Gross morphology**. Photographs of the superficial digital flexor tendon (SDFT) in cross-section at 17 cm distal to the accessory carpal bone (lateral is to the right) and of the palmar surface of the mid-metacarpal SDFT. **A) **fetal-derived Embryonic-like stem cell treated tendons and **B) **placebo control treated tendons. Asterisks mark proximolateral in images of the palmar surface. Click here for file

Additional File 3 **Gene expression and biochemical data**. Selected gene expression and total collagen, proteoglycan and DNA content of fetal-derived embryonic-like stem cell versus placebo control treated tendon following collagenase induction of injury. There were no significant differences between either group for any parameter. Click here for file
