# Supplementary material for: Fetal derived embryonic-like stem cells improve healing in a large animal flexor tendonitis model
Source: Stem Cell Res Ther. 2011 Jan 27;2(1):4. doi: 10.1186/scrt45 (PMC3092144; doi:10.1186/scrt45)
Supplement: Additional File 1 — rt-PCR primer and probe sequences. Sequences (5' to 3') for forward and reverse primers and probes used in quantitative PCR. Sequences were selected from equine specific sequences published in GenBank. [file scrt45-S1.DOCX]

Supplemental Table 1. Sequences (5' to 3') for forward and reverse primers and probes used in quantitative PCR. Sequences were selected from equine specific sequences published in GenBank.

| Gene | Primer | Probe |
| --- | --- | --- |
| *18S* | Fwd: CGGCTTTGGTGACTCTAGATAACC  Rev: CCATGGTAGGCACAGCGACTA | TCGAACGTCTGCCCTATCAACTTTCGAT |
| *COL1A1* | Fwd: GTACCACGACCGAGCCGTAT  Rev: GATCAGGTCATCGCACAACAC | CCGAGCCCTGCCGGGTCTG |
| *COL3A1* | Fwd: CAGGAAGTTGCTGAAGGAGGA  Rev: CCTTCTGGCTTCCAGACATCT | CTCCCATCTTGGTCAGTCCTATGCGGA |
| *COMP* | Fwd: TGGTGGACAAGATTCATGTGTGT  Rev: TGGAAGGCCCGGAAGTC | CGGAGAACGCCGAAGTCACCCTC |
| *DCN* | Fwd: CTGTCCTACATCCGCATTGCT  Rev: TGTAATTCAGTAAGGGAAGGAGGAA | CACCAACATAACCACCATCCCTCCAGGT |
| *MMP1* | Fwd: CATCGTGACAATTCTCCCTTTG  Rev: TACGTGGGCCTGGCTGAA | CTGGAGGAAACCTCGCCCATGCTT |
| *MMP3* | Fwd: CTTATCAAAAATGGCTGCGTCTATT  Rev: AGCAGAGACAGTGTTTTCATTTTTAAG | CCTCTCGGGTAACCCGCTTGTTCC |
| *MMP13* | Fwd: TGAAGACCCGAACCCTAAACAT  Rev: GAAGACTGGTGATGGCATCAAG | CAAAACACCAGACAAATGCGATCCTTCCTTA |
| *SCX* | Fwd: GCGGTGGCAGGACTCTGA  Rev: CCAGAAGAAAACCCAGGTAGGA | AGCACTTGCCCAGACCCACT |
| *TNMD* | Fwd: AAGACTTTGAGGAGGATGGTGAAG  Rev: CCACCCACTGCTCGTTTTG | CTTCCCTACCAACGACAAAAA |
| *TNC* | Fwd: GCCCCTGGCTGAAATCG  Rev: CGGTCACCTGGCAGATCTT | CGGCATCGAGCTCACCTATGGTGTC |
| *SRY* | Fwd: ACAAACGGAGGAGCGGTTAAA  Rev: CTGTTGCAGCGCCGAAT | AGCGTCACAGCAGCACCGCAAC |
