# Supplementary figures and images for: Fetal derived embryonic-like stem cells improve healing in a large animal flexor tendonitis model
Source: Stem Cell Res Ther. 2011 Jan 27;2(1):4. doi: 10.1186/scrt45 (PMC3092144; doi:10.1186/scrt45)

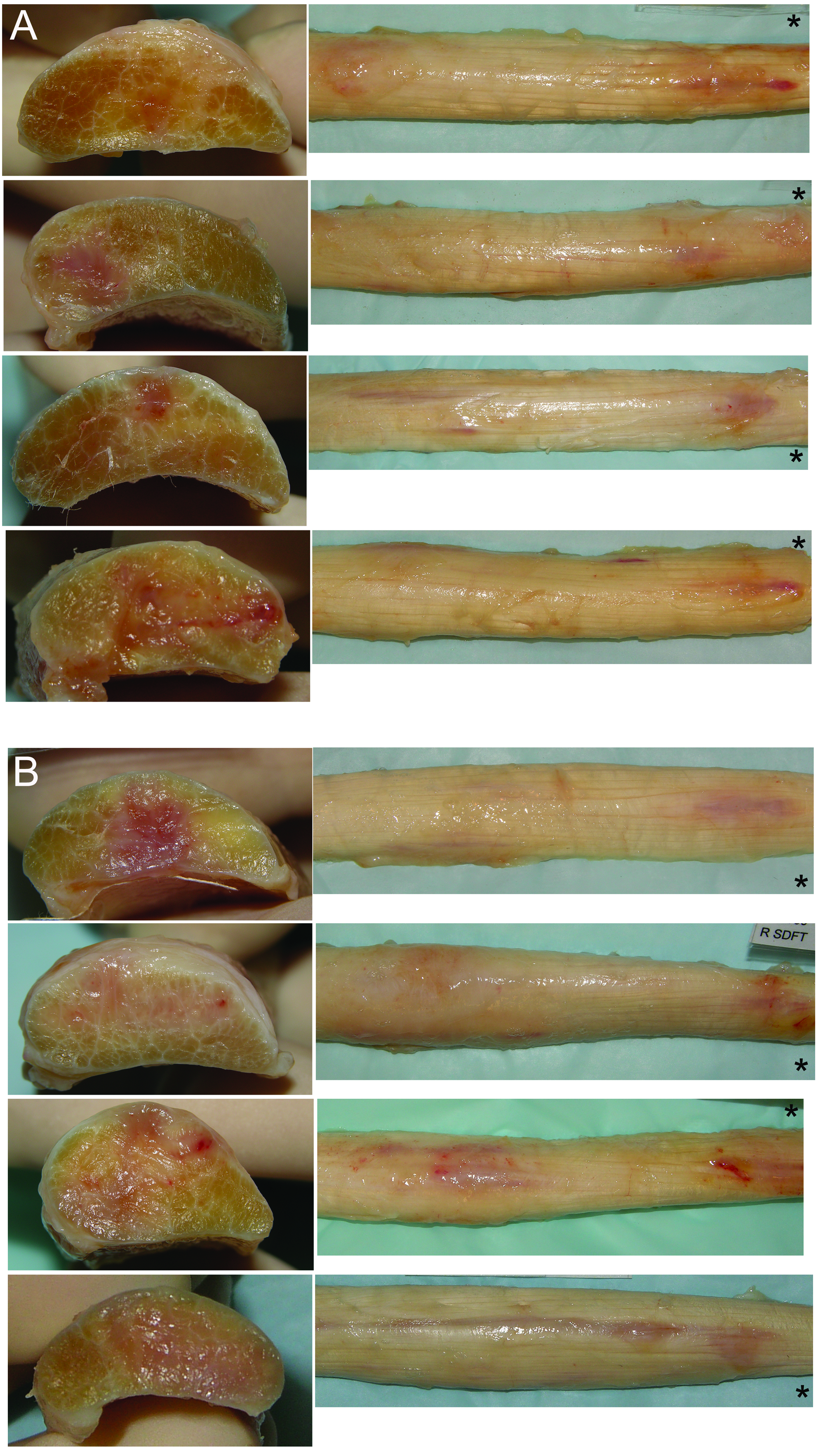

Supplement: Additional File 2 — Gross morphology. Photographs of the superficial digital flexor tendon (SDFT) in cross-section at 17 cm distal to the accessory carpal bone (lateral is to the right) and of the palmar surface of the mid-metacarpal SDFT. A) fetal-derived Embryonic-like stem cell treated tendons and B) placebo control treated tendons. Asterisks mark proximolateral in images of the palmar surface. [file scrt45-S2.TIFF]
