# Supplementary material for: Fetal derived embryonic-like stem cells improve healing in a large animal flexor tendonitis model
Source: Stem Cell Res Ther. 2011 Jan 27;2(1):4. doi: 10.1186/scrt45 (PMC3092144; doi:10.1186/scrt45)
Supplement: Additional File 3 — Gene expression and biochemical data. Selected gene expression and total collagen, proteoglycan and DNA content of fetal-derived embryonic-like stem cell versus placebo control treated tendon following collagenase induction of injury. There were no significant differences between either group for any parameter. [file scrt45-S3.DOCX]

Supplemental Table 2. Gene expression and biochemical data. Selected gene expression and total collagen, proteoglycan and DNA content of fetal-derived Embryonic-like Stem Cell versus placebo control treated tendon following collagenase induction of injury. There were no significant differences between either group for any parameter.

|  | fetal-derived Embryonic-like Stem Cell treated tendon | | Placebo control treated tendon | |
| --- | --- | --- | --- | --- |
|  | Median | 95% Confidence Interval | Median | 95% Confidence Interval |
| Gene expression. Normalized to *18S*. Copy number/ng RNA | | | | |
| *COL1A* | 1.67E+06 | 959,795 - 2,559,000 | 1.80E+06 | 1,319,000 - 2,214,000 |
| *COL3A1* | 338,416 | 136,907 - 545,898 | 375,922 | 195,090 - 559,195 |
| *COL1A*:*COL3A1* | 5.6 | 3.9 - 6.8 | 5.3838 | 2.3 - 7.9 |
| *COMP* | 348,458 | 124,046 - 489,310 | 428,801 | 207,869 - 614,431 |
| *DCN* | 284,205 | 130,104 - 368,565 | 266,690 | 123,900 - 441,629 |
| *SCX* | 25,182 | 11,135 - 35,236 | 28,093 | 21,286 - 36,438 |
| *TNC* | 49,153 | 30,407 - 68,903 | 48,232 | 35,772 - 62,351 |
| *TNMD* | 6,468 | 2685 - 9178 | 6,169 | 2,653 - 8,633 |
| *MMP1* | 438 | 111 - 651 | 54 | -78 – 235 |
| *MMP3* | 100 | -112 - 401 | 15 | 5.9 - 24 |
| *MMP13* | 80 | -76 - 309 | 12 | -43 - 112 |
|  |  |  |  |  |
| Total collagen, glycosaminoglycan and DNA content in µg/mg dry weight of tendon tissue | | | | |
| Collagen | 849 | 694 - 946 | 832 | 650 – 1,044 |
| Glycosaminoglycan | 4.5 | 1.4 - 8.0 | 6.6 | 3.9 - 8.5 |
| DNA | 1.5 | 0.4 - 2.4 | 2.1 | 1.5 - 2.7 |
|  |  |  |  |  |
